# Gendered dimension of chronic pain patients with low and middle income: A text mining analysis

**DOI:** 10.1371/journal.pone.0311292

**Published:** 2024-12-27

**Authors:** Ana M. Peiró, Patricia Carracedo, Laura Agulló, Sónia F. Bernardes

**Affiliations:** 1 Clinical Pharmacology Department, Neuropharmacology applied to Pain (NED), Dr. Balmis General University Hospital, Alicante Institute for Health and Biomedical Research (ISABIAL), Alicante, Spain; 2 Clinical Pharmacology, Toxicology and Chemical Safety Unit, Institute of Bioengineering, Miguel Hernández University, Elche, Spain; 3 Department of Applied Statistics and Operational Research, and Quality, Universitat Politècnica de València, Alcoy, Spain; 4 Universitat Politècnica de València, Valencia, Spain; 5 Iscte-University Institute of Lisbon, Centre for Social Research and Intervention (Cis-Iscte), Lisbon, Portugal; Fundacao Oswaldo Cruz Instituto Rene Rachou, BRAZIL

## Abstract

Inferences about gender differences and socioeconomic status in Chronic Non-Cancer Pain (CNCP) management may shape health care. This can be a particularly relevant issue for women, who continue to more often assume reproductive gender roles. The objective of this study was to explore the gendered impact of CNCP on low-and middle-income women and men through text mining analysis.

**Methods:**

This is a mixed-method study using individual interviews (duration between 40–60 minutes) of 181 CNCP patients (71% females) in a tertiary Pain Care Unit, and applying the text mining methodology. Incomes (low or middle) and gender roles (productive vs. reproductive)”.

**Results:**

Gender differences were identified in the words used to describe pain impact in working and social life, domestic responsibilities, and family relationships. Albeit having similar CNCP severity and interference, women were on average 8 years older, compared to men, with longer referral time from Primary Care, less retired but more homemakers, showing a greater impact on their mental health. The most discriminating word explaining pain impact for CNCP women was “husband”, for men was “work”, especially among middle income groups. The way men, with a productive gender role, talked about the impact of CNCP in their lives stressed the word “work”. In contrast, men with reproductive roles stressed the words “chores, family or limited” as women with low-income did. Only low-income men used the word “help”. The text mining analysis indicates a discrepant distribution of men and women into traditional gender social roles that are consistent with stereotypical traits and may have an impact on pain care. There is a need of an intersectional perspective as part of pain assessment, to develop novel self-management interventions for men and women.

## Introduction

Women are disproportionally affected across a wide variety of chronic pain conditions, including musculoskeletal Chronic Non-Cancer Pain (CNCP) [1, 2] with higher levels of psychological distress [3]. However, compared to men, pain in women is often under-valued being treated less aggressively, generating disparities among patients [4, 5]. These differences reflect a combination of biological and psychological but also, sociocultural factors, making the gender gap in pain patients a complex and widespread problem [6].

This is a particularly relevant issue for women, who continue to take on the largest share of responsibility for family and domestic care despite increasing levels of involvement in the paid workforce [7, 8]. In fact, the long hours required for some of the highest-paid occupations are incompatible with historically gendered family responsibilities. Thus, many professions dominated by women are low paid, and professions that have become female-dominated have become lower paid [9]. This socioeconomic gap is inversely related to health outcomes potentially aggravating gender bias in pain [10]. Moreover, recent studies suggest that women, and individuals of lower socioeconomic status (SES) are more likely to experience more severe pain, and inadequate pain management [11], greater difficulty accessing care, and lower satisfaction with pain care [12, 13]. This pay gender gap can be another added barrier to achieving equity in pain care, aggravating gender bias [14].

Much work has been done to think critically about sex and gender impact—behavioural norms applied to males and females that influence societal expectations, and experiences- on health behaviours and outcomes [15]. Here, we studied CNCP patient’s language through text mining analysis, trying to wonder its cognitive reflection from textual data. Text mining analysis–that combines data mining, linguistics, computational statistics, and computer science- offer a way to quickly extract high-quality information from unstructured text [16, 17]. This could help us to understand the ways in which women and men communicate pain [18, 19] by uncovering sex- related differences in the language used [20]. For this reason, text-mining was used to analyse the gendered impact of CNCP on low and middle incomes women and men patients using structured interviews. Briefly, we use the statistical text mining technique to analyze patient testimonies and detect significant behavioural patterns related to gendered impact of CNCP on low-and middle-income women and men.

## Materials and methods

### Study design and participants

A mixed-methods (quantitative and qualitative) cross-sectional study was designed and performed at Pain Unit (PU) of the Alicante Health Department–Dr. Balmis General University Hospital, in Spain, from October 2021 to July 2022. A total of 181 patients with CNCP (71% women) were included to provide quantitative and qualitative information. Inclusion criteria were adults (≥18 years), with CNCP who have required opioid analgesic treatment, and consent informed. Patients under 18 years old, with oncologic pain or any psychiatric disorders that could interfere with the proper development of the study were excluded. Other chronic pain syndromes of unclear pathophysiology, as fibromyalgia, or neuropathic pain as painful polyneuropathy, postherpetic neuralgia, trigeminal neuralgia, and post-stroke pain were not included.

### Procedure and data collection

A consecutive sampling method was used in ambulatory patients. The researchers reviewed the schedule of PU cited patients, one day a week, usually on Thursdays and prepared the questionnaires and informed consents. When a patient met the inclusion criteria, he/she was informed about the purpose of the study by the PU healthcare team. Afterward, interested individuals were attended by the research staff to sign the informed consent and collect all variables. If needed, they were completed using Electronic Health Records (EHRs), which allows reviewing medical diagnoses, outcomes and medication use.

### Clinical outcomes

A Global Pain State questionnaire measuring quantitative pain intensity, relief and quality of life was collected at the time of the interview. Pain intensity and relief were measured using a Visual Analogue Scale (VAS). Both consist of a horizontal line ranging from 0 (lowest) to 100 mm (highest), where the patient points on the line to the intensity of pain or relief that he/she feels, respectively. Other demographic characteristics such as age, sex, gender identity, employment status (active, retired, work disability, unemployed or homemaker) were also registered.

Quality of life was evaluated with the EuroQol-5D-3L scale (53112 reg. number available at https://euroqol.org/) that consists of a VAS (vertical line from 0 mm (the worst imaginable health status) to 100 mm (the best imaginable) where the patient indicates his/her actual health status. Health utility status (0 death to 1 perfect health, using dimensions of mobility, self-care, usual activities, pain/discomfort, and anxiety/depression) was calculated [21]. Short Format Health Survey 12 (SF12, 12-item questionnaire with a mental (MCS, 19–61 scores) and a physical component score (PCS, 24–57 scores) with a mean of 50 and a standard deviation of 10 in the general U.S. population [22] was also used. Psychological status was calculated due Hospital Anxiety and Depression Scale (HADS, 0–21 scores, comprises seven questions for anxiety and seven questions for depression). Here a score of 8 or more for anxiety has a specificity of 0.78 and a sensitivity of 0.9, and for depression a specificity of 0.79 and a sensitivity of 0.83 [23].

### Pharmacology and hospital resource use

The use (yes/no) of analgesics, non-steroidal anti-inflammatory drugs, and opioids were registered. In different opioids’ combinations, oral morphine equivalent daily dose (MEDD) was estimated using available references [24]. Number of adverse events (AEs) were collected with a list of the most frequent analgesic side-effects from the Summary of Product Characteristics. Hospital admission, emergency department visits, and drug change prescription were registered. Time gap from first diagnosis associated with pain, until referral to PU (range: < 1 year, 1–2 years, 2–5 years, and > 5 years) was calculated.

### Quantitative and qualitative gender information

The sources of information were a semi-structured interview administered to patients developed in 2018 by the Center for Biomedical Research in Epidemiology and Public Health Network (CIBERESP) in collaboration with Rheumatology Department from Alicante University General Hospital (Spain) (S1 Appendix). Three trained interviewers conducted face-to-face interviews that lasted 30 to 45 minutes. Quantitative information was obtained by collecting yes/no answers to the 15-question related to gender (roles, identity) as can be seen at **S1 Appendix**. Here, questions number 2, 3,4, 5 and 6 are related to gender identity (as a woman/man?) whereas “gender roles” are related to 7 (work), 9 (domestic responsibilities), 11–12 (partner relationships) and 13 (family). The percentage of affirmative answers to each question was compared between sexes grouped into high level impact (> 7 affirmative answers).

Each patient was identified as "Women" or "Men" and a consecutive number was assigned. Some participants’ speeches were selected to illustrate sex-related differences showed by text mining analysis. A preliminary selection from data base was done by L.A. and A.P. separately; which was later reached by consensus.

### Text mining analysis

In this context, an open-ended question is a major element of text mining that constitutes a group of discrete textual data within a collection. Given the technical requirements of the method used, the database must be processed to avoid terms that could distort the results. Next, we show step by step the actions necessary to process the testimonials for analysis: 1 remove redundancies by ignoring case, 2 delete punctuation marks, 3 remove digits at the end delete stop words, which are commonly used in a language but that do not provide information in the text analysis. Some examples in English are that, then, the, a, an, and, among others.

## Correspondence analysis

Like numeric dimension reduction techniques, text mining abridges outliers, low frequency phrases and important information. Correspondence analysis (CA) is a multivariate statistical technique for visualizing and describing the relations between two or more variables. Specifically, it explores the relationships between words (terms) and documents (texts) within a large corpus [25].

It is particularly applicable to a table of categorical but can also be used to visualize non-negative data or a common ratio scale. The methodology followed involves the normalisation of the cross-table of frequencies, so that the cross-table entries can be represented in terms of distance between dimensions in a low-dimensional space. Its main objective is to represent the rows and columns of a data matrix as points in a spatial representation called a map or a biplot [26, 27]. The positions of the points suggest a facilitate interpretations of the data content [28]. Specifically, each word is represented by a point, with the distance to the closest one indicating the similarity between them [29].

The distance between words and the origin measures the quality i.e. words that lie farther from the origin will be more discriminatory and well represented on biplot [30].

### Statistical data

The sample size calculation was not performed because a convenience sample was used in this study according to regular attendance of patients to their clinical visits. This entailed selecting participants on the basis of availability until the final sample size was achieved.

A descriptive analysis of continuous quantitative variables (i.e., pain intensity and pain relief, quality of life, health utility status and SF12 scores) was presented as mean and standard deviation (SD), and discrete variables (i.e., age, HADS scores and AEs) are shown using their median and interquartile rage (IQR), while categorical data (sex, employment status, anxiety and depression groups, pharmacological prescription, gap time interval, and quantitative gender response data) was expressed by percentages. Strobe guidelines suggest that inferential summaries should be avoided in descriptive tables for observational studies.

Statistical analyses were carried out using R software package 4.03 and GraphPad Prism software 5.0. Data were extracted from the full text of interveiwers by P.C. Data extracted included the following items from the Gender questionnaire, Sample characteristics (e.g. participants, age, sex, level of education), Clnical and Pharmacology Data, Approaches to data analysis (e.g. pre‐processing and statistical methods), Key themes.

### Ethics statement

This study was approved by the Ethics Committee Board of Dr. Balmis General University Hospital of Alicante (code: 2023–0130). Participants gave verbal and signed informed consent before participating in the interviews, and confidentiality of all information was guaranteed. The study conforms to the Declaration of Helsinki regarding research involving human subjects. The datasets generated are available from the corresponding author on reasonable request.

## Results

### Sex related differences in CNCP

A total of 181 participants were included, mostly Caucasian middle-aged (63 [52–73] years old) women (71%) residents in Spain. A summary of sex related differences in clinical and pharmacological outcomes is presented in **Table 1.**

**Table 1:** Socio-demographic, clinical and pharmacologic data by sex in chronic non-oncologic pain population. Values are %, mean (SD) or median [IQR].

				Total (n = 181)	Women (n = 129)	Men (n = 52)
Sex (%)				181 (100%)	129 (71.27%)	52 (28.73%)
Age (years old)				62.13 (13.57)	63.45 (13.69)	58.74 (12.77)
				63 [52, 73]	65 (53, 75)	57 (50.25, 69)
Employment status (%)						
Active			18		17	18
Retired			37		33	49
Disability/ Unemployment			29		28	33
Homemaker			16		22	0
Monthly incomes (euros)						
> = 1000 euro			36		33	44
<1000 euro			43		60.5	40
NA					6,5	16
Pain intensity (0–100 mm)			70.35 (26)		70 (26.83)	71.25 (23.96)
			80 (50, 90)		80 (50, 90)	72.5 (58.75, 90)
Pain relief (0–100 mm)			36.6 (29.87)		39.3 (30.56)	29.79 (27.17)
			40 (0, 60)		45 (0, 60)	25 (0, 50)
Quality of life (0–100 mm)			46.43 (25.81)		45.66 (24.19)	48.38 (29.67)
Health Utility Status (0–1)			0.25 (0.05, 0.65)		0.25 (0.05, 0.65)	0.26 (0.05, 0.58)
Physical C. (SF12)			27 (7.5)		27 (7)	27 (9)
Mental C. (SF12)			41 (12)		40 (11)	45 (13)
HAD–Anxiety	Negative		42.5		45	61
Doubt			18		22	17
Case			25		32	22
HAD–Depression		Negative	51		59	61
Doubt			16		21	12
Case			19		20	27
MEDD (mg/day)			60 (30, 99.45)		60 (30, 102.3)	60 (37.5, 80)
Simple analgesics			46		50	38
Neuromodulators			44		45	40
Antidepressants			35		33	42
Anxiolytics			42.5		44	38.5
Health Resources Use data (%)						
Emergency Department Visits			26.5		28	23
Hospitalisation			5.5		5	6
Prescription Change			18		17	19
Diagnosis Delay						
< 1 year			19		15.5	27
1–2 year			12		11	15
2–5 year			18		19	15
> 5			32		38	19

Women were on average 8-years older than men, with a different employment status being higher homemaker and less retired, in front of 49% of men. Any of the men reported being homemakers. Women with lower incomes (< 1000 eur/month) was higher than men. In contrast, 10% more men did not refer their employment status in front of women.

Clinical status was similar with a mean of severe pain of 70 (SD = 26) mm, moderate quality of life 46 (26) mm, and low health utility status, under a similar mean opioid dose (60 mg/day). However, mental component of SF12 was higher in women, as well as anxiolytics prescription. Women present a higher diagnosis delay to Pain Unit remission from Primary Care, from first diagnosis in EHR.

## Sex related differences in word use in CNCP patients

**Fig 1:**
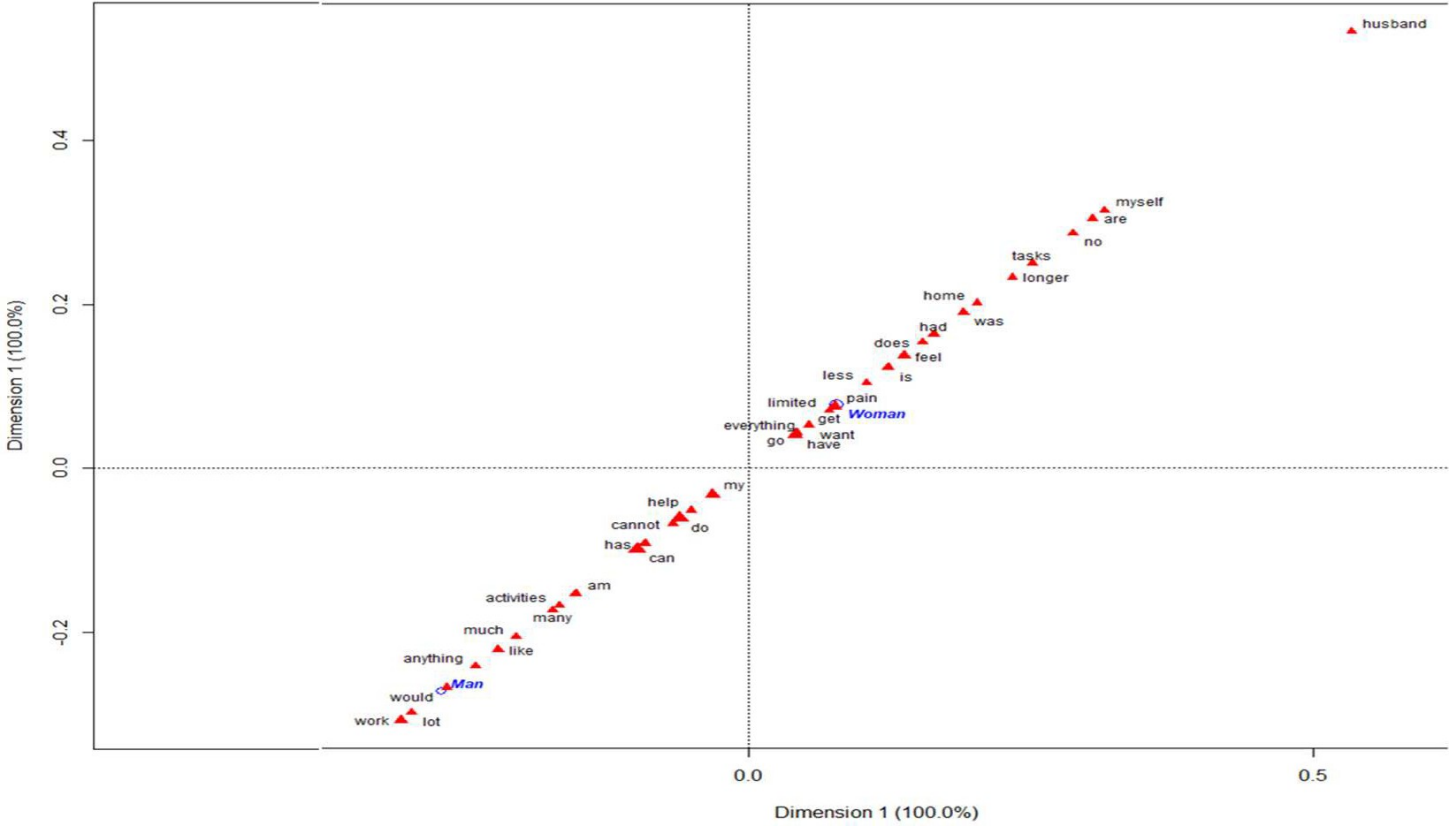
The first plane of the correspondence analysis with most contributive terms by sex.

**Fig 1** represents the most frequent words used (red triangle) by sex on a two-dimensional plane, where the size of the triangle indicates the frequency of the word. Terms that are closer to the origin (0,0) will be less discriminative, while those further away will be more discriminating. Closeness between terms (groups) indicates similarity (clusters). In the case of women, the word that carries the most weight was “husband” followed by “myself”, “task”, or home”. In contrast, men used “work” followed by “activities” or “like”.

Some speeches that can illustrate these sex-related differences are: Women indicate that *they can’t do anything at home*, *due pain*, *and all the time they need help to do everything*. Thus, even if the woman is on pain, *she has to be responsible for housework and paid work*, *even if she asks her husband for help*, *she feels bad*. *I must make meals because my husband works late*, *and someone has to feed our children*.

Regarding men, *they have had to stop working and feel limited*, *given up many activities with friends because of the limitations of pain*. Because *men can’t do many things they feel helpless and have to ask for help*. To avoid dismissal, some women *conceal their limitations and prioritize their work over their self-care*. *Men seem to be more affected in certain jobs that require optimal physical condition*.

## Gender role impact in CNCP men and women

**Fig 2:**
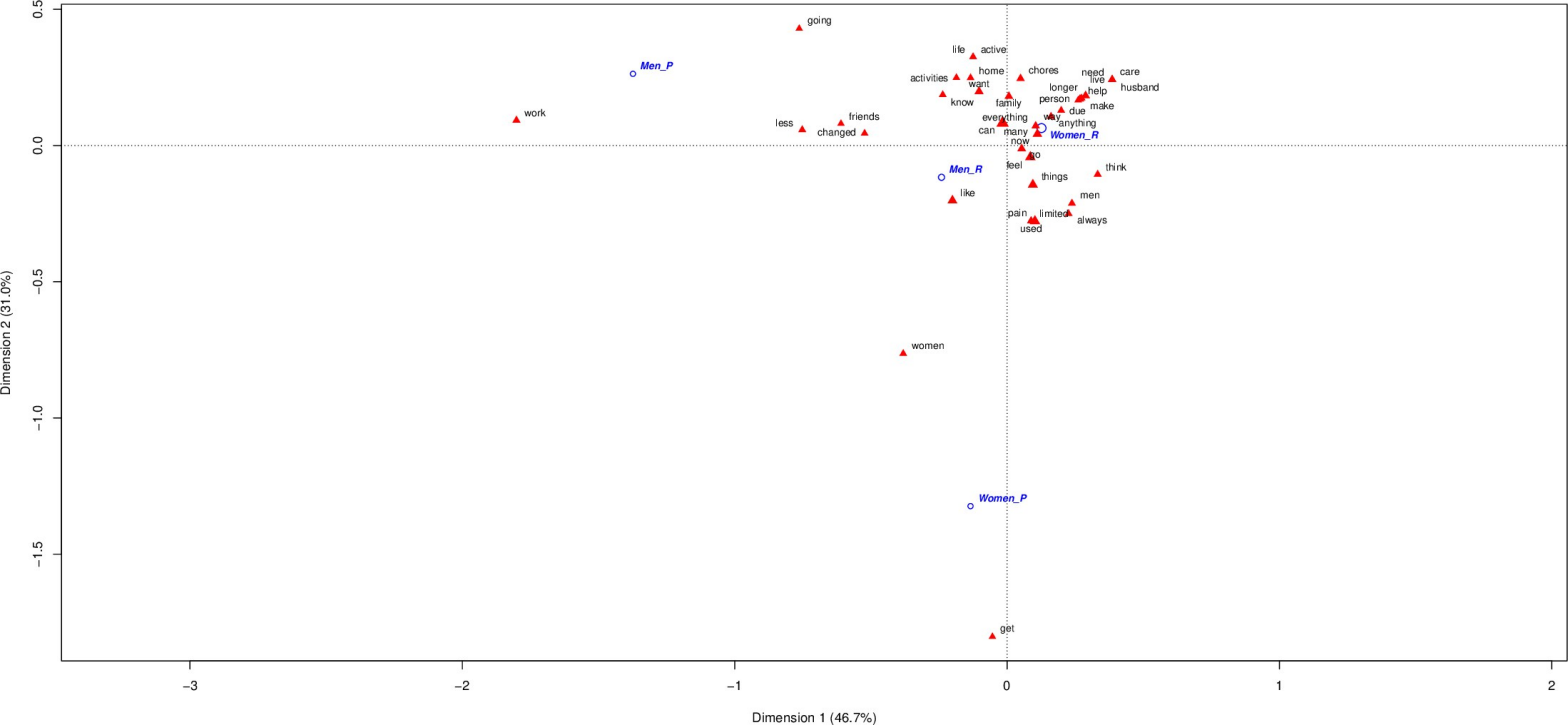
The first plane of the correspondence analysis with most contributive terms by sex and role.

**Fig 2** includes sex (women, men) and gender role (productive [P], reproductive [R]). In the case of paid-work men, the most significant words are “disability” and “work”; and for paid-work women “get” and “women”. In contrast, men or women on reproductive role shared similar words as “chores, family, pain, or limited”. The most discriminatory word for women- on reproductive gender role- continue being “husband” *(My husband would have died if he had to endure my pain*. *I worry about my family and husband because I can’t’ keep carrying and providing for them*. *This doesn’t help the family dynamic*, *and worsens when is time to come back*. *My husband doesn’t help has much as he could with the house chore)*.

In contrast, for men -without productive or reproductive roles, most significant words is “home” *(I´ve been at home more*, *and been on a limited number of outings*.*)*. No men talk about “wife” however men with less stereotypical reproductive gender role, refer to “men” *(Because the chores performed by women differ from those of men*. *Because men care less about housework*.*)* in the same way that women under a paid work that use “women”. They more often refer than themselves, as belonging to this group.

Men with productive role is in the extreme word use of men with reproductive role, nor with women. In general, there are more and more complex narratives in the population with reproductive roles. However more focus on “themselves” with words as “men, things, use”. Women on reproductive roles are more focused on the others “husband, chores, home, family” *(I think that women shoulder more responsibilities "house*, *family*, *work"*. *I have my partner’s family*, *but don’t have the strength to meet up with them)* as identity treats; as a caregiver and less focussed on their disability or work. There is less contrast between women roles in front of men, that are in opposites clusters due gender roles.

## Incomes impact in CNCP men and women

**Fig 3:**
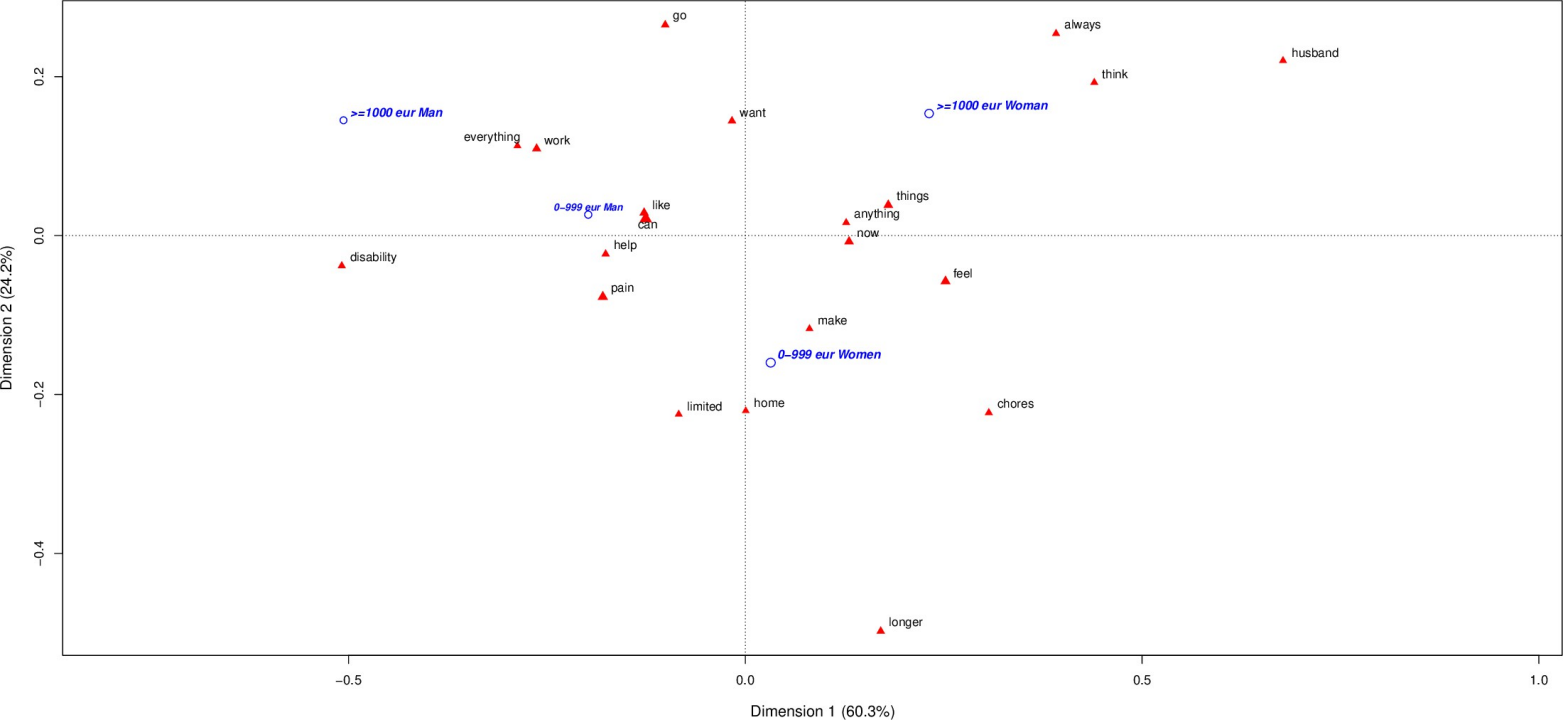
The first plane of the correspondence analysis with most contributive terms by sex and incomes.

**Fig 3** includes sex (women, men) and incomes (less or more 1000 euros). Lower income men are focused on “jobs disability and help”, medium paid men are focussed on “work”. Women lower incomes are focused on “chores and homes”. In contrast, incomes > 1000 euro/month ones are focused on “husband, things”. In really excluded people, women continue with the trait related to caregiver and men with being “disable” and “help”.

## Discussion

This study aimed to examine the gendered impact of CNCP using text mining analysis of structured interviews. Common themes were the functional limitations caused by pain, more related to work and disability, especially in lower incomes. Moreover, domestic gender roles continue being identificative for women—under a different employment status and lower incomes who showed a significant higher pain delayed CNCP referral to PU with a higher mental health impact, in front of men. Our findings in real-world ambulatory CNCP patients suggest that pain-related gender stereotypes may still being an obstacle to an early and equal pain care access for women.

The study indicates that while household chores may have changed in the past decades, the socioeconomic gap is able to aggravate gender pain inequalities in pain care [31]. Moreover, our results showed a possible gender bias in CNCP Primary Care referrals for PU management being women more vulnerable to wait longer showing a higher mental health impact, in front of men. Women experienced pain for many years, they sensed a lack of understanding from others, they were fearful of what might be wrong with them [32]. It is a reality that low gender sensitivity and high sexism can be potential target for intervention as modifiable risk factors for health inequities in pain care [33, 34]. Experimental approaches to characterizing gender biases could inform the development of interventions to reduce them improving pain management equity [35].

Our results showed that paid work men used fewer words, less descriptive language, and focused on work and themselves. In contrast, men with lower incomes or far from traditional domestic gender roles beliefs, used more complex narrative and ask for help. Increased knowledge of socioeconomic risk factors for long term pain, e.g. low employment status or incomes, is needed on all levels within the healthcare system in order to facilitate effective communication in the treatment of pain and potential gender bias in pain [36]. The potential socioeconomic gap (higher low-incomes women worried for their *husbands* and higher paid-men about *work*) can aggravate, as in other studies, the legitimation of pain, the willingness to offer support, and credibility only in women who, sometimes struggling to explain their inability to maintain work and home responsibilities [37]. All of them could link to a stereotyping healthcare professional’s performance in pain cares as in other chronic diseases [38, 39].

Results could lead in unconscious biases and almost instinctive preferences and behaviours that may favour men, in pain care [40]. Thus potential social factors can contribute to the differences observed in our study. Here, we propose three key areas of education and research that should be prioritised in order to address the unmet needs of women under CNCP as in other widespread pain [41]: (1) to identify ways to increase awareness of pain occurrence in women patients among healthcare professionals trying to avoid diagnosis delay and mental impact (2) to improve understanding of gender differences in CNCP worries due gender roles or incomes, and (3) to conduct gender-stratified clinical trials with a representative sample of women patients. An overall risk-profile in terms of psychosocial and biological factors needs to be assessed early on within pain management due sex and gender [42].

## Limitations

There are some limitations in this study that need to be acknowledged. First, the sample size was limited by a “convenience sample” with similar demographics (white, middle aged) included in a single hospital. In fact, the female predominance can reflect the higher CNCP prevalence in real-world patients [43, 44]. Therefore, the results of the present study may not be representative of the experience of the wider population of men and women with CNCP. Second, the results are based on qualitative testimonies and the gender questionnaire is a new scale that needs to be validated in other heterogeneous populations. Third, due the median age of the sample, the number of patients in active employment is very low, thus sex differences are difficult to compare. Consequently, the results of this study may have been affected. In fact, socioeconomic status is based on economic situation, education and occupation [14] and our study only analysed incomes. Finally, there are other important factors that were not controlled during the study, such as the women predominance, the duration of pain, other variables as family members or social support and that the financial situation can be a variable that could change along life-spam. All of them could interfere with the occurrence of pain experience. All of this should be taken into account in future analysis.

Text mining suggests a discrepant distribution of men and women into traditional gender social roles that could influence on pain management. Significant different behavioural patterns have been observed related to gender and incomes in CNCP real world patients that could influence on pain management due higher pain delayed CNCP referral to PU and mental health, in women. Therefore, sex and gender analyses need to be performed to develop personalized pain care.

## Supporting information

S1 Appendix Questionnaire to patients (English version). (DOCX)
