# Supplementary material for: Gendered dimension of chronic pain patients with low and middle income: A text mining analysis
Source: PLoS One. 2024 Dec 27;19(12):e0311292. doi: 10.1371/journal.pone.0311292 (PMC11676948; doi:10.1371/journal.pone.0311292)
Supplement: S1 Appendix — (DOCX) [file pone.0311292.s001.docx]

**Supplementary Material (S1 Appendix)**

**Questionnaire to patients (English version)**

1. Has your pain changed the way you are? Yes / No How?
2. Has the pain affected your self-esteem as a woman/man? Yes / No How?
3. Has the pain changed your image of yourself as a man/woman? Yes / No How?
4. Has the pain changed your masculinity or femininity? Yes / No How?
5. Has the pain generated a conflict between what you want/can (do) and what you think your family environment asks of you as a woman/man? Yes / No How?
6. Has the pain generated a conflict between what you want/can (do) and what the social environment asks of you as a woman/man? Yes / No How?
7. Has the pain affected your work tasks and/or responsibilities within your work environment? Yes / No How?
8. Did you do household chores before the diagnosis of the disease? Yes / No
9. Has the pain affected your tasks and/or domestic responsibilities? Yes / No How?
10. Has the pain affected your life project or your future plans? Yes / No How?
11. Has the pain affected your relationships? Yes / No How?
12. Has the pain affected your sexual relationships? Yes / No How?
13. Has the pain affected your family relationships? Yes / No How?
14. Do you think that your social, work or family position has worsened due to the pain? Yes / No How?
15. Do you think that the experience of pain would have been different instead of a man being a woman (or vice versa) ? Yes / No How?
